# Insights into metabolic characteristics and biological activity changes in Zangju (*Citrus reticulata* cv. Manau Gan) peel at different maturity stages through UPLC–MS/MS-based metabolomics

**DOI:** 10.1016/j.fochx.2024.101197

**Published:** 2024-02-05

**Authors:** Peng Wang, Haifan Wang, Yang Xiao, Jialiang Zou, Hongping Chen, Lin Chen, Fu Wang, Yuan Hu, Youping Liu

**Affiliations:** a Department of Pharmacy, Chengdu University of Traditional Chinese Medicine, Chengdu 611137, China; b State Key Laboratory of Southwestern Chinese Medicine Resources, Chengdu 611137, China

**Keywords:** *Citrus reticulata* cv. Manau Gan, Nontargeted metabolomics, Maturity stage, Differentially accumulated metabolites, Biological activity

## Abstract

In this study, comprehensive and systematic nontargeted metabolomics analysis was performed with the metabolites of Zangju peel (*Citrus reticulata* cv. Manau Gan, CRZP, which has been cultivated for over 400 years in Derong County, China.) at four different mature stages. A total of 1878 metabolites were identified, among which flavonoids were the most abundant (62.04 %), and identified 62 key differential metabolites significantly affected by maturity. Based on biological activity measurements, CRZP showed better antioxidant activity, lipase inhibition ability, inhibition of adipogenic differentiation in 3TT-L1 cells and promotion of lipid metabolism, with the biological activity of CRZP at different maturity stages being associated with key differential metabolite. Thus, CRZP is natural antioxidants and possess anti-obesity potential, and industrial production needs to consider the Maturity stage of its collection.

## Highlights

• The 1878 metabolites have been identified, which flavonoids were the most abundant.
• The CRZP showed strong antioxidant activity.
• The CRZP has good pancreatic lipase inhibition and cellular lipid-lowering activity.
• The maturity had greater impact on the metabolites profiles and biological activity.

## 1 Introduction

Citrus fruits, with their unique flavor and nutritional value, are among the most consumed fruits worldwide and are grown in over 140 countries and regions globally (FAO Statistics, 2021; https://www.fao.org/statistics/en/). Previous studies have shown that modern citrus is derived from two hybrids (e.g., sweet orange, *C. Unshiu*) or three hybrids (lemon) of its ancestors (*C. reticulata*, *C. maxima*, *C. medica*), followed by various locally cultivated varieties (e.g., *C. reticulata* ‘ChaChi’, *C. reticulata* ‘ponkan’) due to human intervention (Wu et al., 2018), which are important in industrial production. In addition to being consumed as fruits, citrus is also processed and used to produce other commodities, such as juice/juice drinks, preserved fruits, and canned fruits. However, the production of citrus peel waste after processing is approximately 50–70 % w/w of citrus fruits, depending on the technology used and the variety of fruit cultivation, and its annual global production may be close to 10 million Mg (Mg is the SI unit equivalent to tons) (Matsuo et al., 2022, Zema et al., 2018). The medicinal use of citrus peel can be traced back to the 10th century, and its bioactive components have only recently been characterized. Citrus peel is rich in polyphenolic compounds, including flavonoids, limonoids, terpenoids, lignans, coumarins, phenolic acids, and other compounds. The peel exhibits anti-inflammatory, antioxidant, anticancer, and lipid-lowering effects and has been used as a source of functional active ingredients in food, medicine, spices, and other fields (Gomez-Mejia et al., 2019, Rafiq et al., 2018, Tian et al., 2018). However, research on the peel of different citrus varieties is limited, especially in locally cultivated varieties, leading to the waste of industrial byproduct resources. More research and knowledge are needed to fully utilize the peel of different citrus varieties.

In China, local varieties of *C. reticulata* are abundant, such as *C. Unshiu*, *C. reticulata* ‘Chachi’, *C. reticulata* ‘Ponkan’ and Zangju (*C. reticulata* cv. Manau Gan). Their dried fruit peels (CP) are often used as medicinal, health food, or food seasoning agents (e.g., ‘Preserved Mandarin Peel’- preserved fruit, ‘Ganpu Tea’ - using *C. reticulata* ‘Chachi’ and Pu'er tea as raw materials, Tangerine Powder) (Lv et al., 2020). However, the peel byproducts of many local varieties of citrus are not comprehensively utilized. Among them, Zangju peel (CRZP) is used by local residents as a cooking season due to its unique aroma and flavor and is often added to beef and mutton stews to reduce the greasy taste. Zangju is known locally (in the Tibetan region) as “Jia Xu”, symbolizing “fruit of longevity”; this name may be inspired by the oldest Zangju tree in the area, which has been growing for a hundred years. Zangju fruits are popular among people because they generate a unique flavor and are easy to peel. The fruit is mainly distributed in Derong, Muli and other counties in the agricultural and pastoral areas of the middle and lower reaches of the Sanjiang River Valley (subtropical valley climate) in the southern section of the Hengduan Mountains, China (Song, 2016). The fruit has been cultivated for over 400 years, with a planting area of over 11,600 ha and an annual output of approximately 130,000 tons (according to local government statistics, https://www.nongjixie.org/Library). Although there have been numerous studies on the peel of *C. reticulata* local varieties (Costanzo et al., 2022, Luo et al., 2017, Wang et al., 2022), no research has been performed on the CRZP. Thus, the potential of CRZP is an attractive topic to investigate in detail, especially its metabolic characteristics and changes in biological activity during maturation, as this variety represents a typical resource in many underdeveloped citrus local varieties in China. In our previous research, we identified the volatile compounds of CRZP and their changes at different ripening stages, including alcohols and aldehydes that produce a green fruit aroma, as well as monoterpenes, ketones, and esters with a mature fruit aroma. We also screened potential flavor markers to distinguish different stages (Wang et al., 2023, Wang et al., 2023). As shown by previous studies, in addition to using citrus peel as an essential oil extraction and feed source (Bampidis and Robinson, 2006, Calabrò et al., 2016), citrus peel could provide a source of phytochemicals as a supplement in the human diet; thus, there is enormous development potential and application prospects for citrus industrial byproducts (Deng et al., 2012, Elkhatim et al., 2018, Manthey and Grohmann, 2001), and maturity should be an important parameter to evaluate the application potential of citrus peel (Costanzo et al., 2022, Moulehi et al., 2012). During the ripening process of citrus fruits, the color of the citrus peel changes from green to orange red or bright red, and the corresponding content of polyphenols, carotenoids, flavonoids, and other compounds changes (Maduwanthi and Marapana, 2019, Multari et al., 2020). In addition to maturity, different varieties, cultivation methods, climate conditions, etc., also affect the final characteristics of citrus fruits (Moulehi et al., 2012). Therefore, evaluating the metabolic characteristics and changes in biological activity of CRZP during the maturation process is crucial for using CRZP as a functional food and gaining a deeper understanding of its chemical composition characteristics and functional activity.

In recent studies on food ingredients, nontargeted and targeted metabolomics analysis based on liquid chromatography–mass spectrometry (LC–MS/MS) has been widely applied (Cheng et al., 2020, Lyu et al., 2021, Miao et al., 2023). Compared to traditional liquid chromatography (HPLC), nontargeted metabolomics can separate components and utilize MS characterization to identify as many metabolites as possible (Bach et al., 2018, Song et al., 2019). In particular, MS/MS can provide structural information from rich fragment ions, and retention time can provide further evidence. Generally, the method can cover common metabolites with moderate polarity in plants and be used to explore the chemical characteristics of unknown objects. However, quantitative analysis of the main active ingredients is lacking in nontargeted metabolomics, and in this case, selecting targeted metabolomics is a suitable strategy. For example, when the active components in experimental materials are known, HPLC can be used to perform quantitative analysis with the main flavonoid compounds naringin, melididin, and neoepicitrin in Shatianyu (*Citrus grandis* L.) (Deng et al., 2023). Overall, adopting a nontargeted metabolomic research strategy can more effectively characterize the metabolic characteristics of foods with unknown components.

The aim of this study was to evaluate the potential and possibility of utilizing CRZP as a natural functional food source, as well as the impact of maturity on the metabolic characteristics and biological activity of CRZP. Therefore, the experimental design of this study was performed as follows: (1) nontargeted metabolomics based on UPLC–MS/MS was used to characterize the chemical characteristics of CRZP and analyze the differential metabolites of Tibetan orange peel at different maturity stages; (2) differences in the biological activity of CRZP collected at different maturity stages were analyzed; and (3) the correlation between key differential metabolites and biological activity were analyzed and the changes in chemical composition and biological activity during the maturation process were revealed.

## 2 Materials and methods

### 2.1 Plant material

**Fig. 1:**
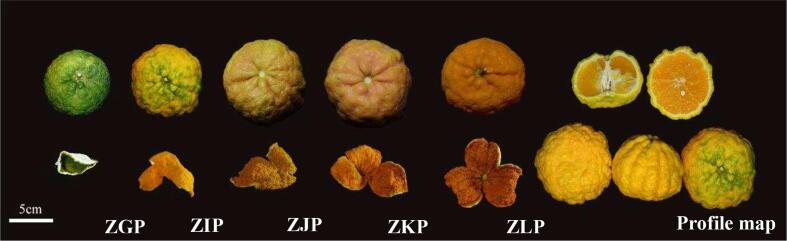
Zangju (*Citrus reticulata* cv. Manau Gan) samples from each stage.

The samples (*C. reticulata* cv. Manau Gan, Zangju) were all obtained from Derong County, China (99° 16′ 37′′ E; 28° 32′ 32′′ N; 2225.15 m); 4 adjacent Zangju trees with high yield and stable quality were randomly selected, numbered and listed, and 6 fruits of similar size and normal growth were randomly selected from each tree. Samples once a month (from October 2022 to February 2023, the same growth conditions) were numbered ZGP, ZIP, ZJP, ZKP, and ZLP, as shown in Fig. 1. We divided the 5 harvest stages into 4 mature stages based on their appearance and color characteristics. The immature stage (October) has a dark green peel, the immature stage has a yellow mixed with green peel (November), the mature stage has an orange peel (December), and the fully mature stage has an orange peel (January and February of the following year). The collected samples were washed, the skin was removed manually, and the samples were dried with hot air at a constant temperature of 50 ℃ for 18 h. After preparation, all samples were stored in a dry place at room temperature. The processing and storage of samples after collection are carried out in the science and technology building of the department of Pharmacy, Chengdu University of Traditional Chinese Medicine.

### 2.2 Chemicals and reagents

3T3-L1 preadipocytes, dulbecco’s modified eagle’s medium (DMEM, high glucose), Fetal bovine serum (FBS) were purchased from Procell Life Science&Technology Co.,Ltd.. Methanol, acetonitrile (Fisher Chemical, USA). Formic acid (MREDA Technology Inc., USA), all of which were LC–MS grade. Reactive oxygen species (ROS) assay kit (Beyotime Biotechnology Co., Ltd), Cell Counting Kit-8 (CCK-8, BOSTER Biological Technology co.ltd), Total antioxidant capacity (*T*-AOC) assay kit (Ferric ion reducing antioxidant power, FRAP), Superoxide dismutase (SOD) assay kit, Malondialdehyde (MDA) assay kit, Glutathione peroxidase (GSH) assay kit, nonestesterified fatty acid (NEFA), glycerol assay kit, Oil Red O and BCA protein assay were purchased from Nanjing Jiancheng Biotechnology Research Institute, China. Pig pancreatic lipase (30,000 u/g, Shanghai Yuanye Biotechnology Co., Ltd., China). Triiodothyronine (T3), dexamethasone, isobutylmethylxanthine (sigma), indomethacin and rosiglitazone were purchased from Shanghai Aladdin Biochemical Technology Co.,Ltd.. Insulin, 2,2-Diphenyl-1-Picrylhydrazyl (DPPH) and 2,2′-azino-bis(3-ethylbenzothiazoline-6-sulfonate) (ABTS) were both purchased from Shanghai Macklin Biochemical Technology Co., Ltd. China, 4-nitrophenyl laurate (purity ≥ 98 %, Beijing Jingming Biotechnology Co., Ltd. China), Tris base, Servicebio, anhydrous sodium acetate and hydrochloric acid (purity ≥ 98 %, Chengdu Kelon Chemical Co., Ltd., China) were both AR grade.

### 2.3 Sample preparation and extraction

The dried peels of oranges from different ripening periods were ground with a grinder (MM 400, Retsch) at 30 Hz for 1.5 min until they were a powder. Fifty milligrams of powder was accurately weighed and added to 1.2 mL of a precooled 70 % methanol solution at −20 ℃. The mixture was vortexed once every 30 min for 30 s, for a total of 6 times. After centrifugation (at 12,000 rpm for 3 min), the supernatant was extracted, a microporous filter membrane (0.22 μ Filter the sample) was used, and the filtrate was stored in the injection bottle for UPLC–MS/MS analysis. Quality control samples (QC) were made by mixing five sets of sample extracts.

### 2.4 UPLC–MS/MS conditions

The UPLC–MS/MS system for analysis mainly includes ultra-performance liquid chromatography (UPLC) (ExionLC ™ AD, https://sciex.com.cn/) tandem mass spectrometry (MS/MS, SCIEX Triple Quad ^TM^ 6500+). Chromatographic conditions: (1) Chromatographic column: Agilent SB-C_18_ 1.8 µm, 2.1 mm * 100 mm; (2) Mobile phase: A phase was ultrapure water, B phase was acetonitrile, both of which were added with 0.1 % formic acid; (3) Elution gradient: The B phase ratio was 5 % at 0.00 min, and linearly increased to 95 % within 9.00 min and maintained at 95 % for 1 min, 10.00–11.10 min, and the B phase ratio decreased to 5 % and equilibrated at 5 % to 14 min; (4) Flow rate 0.35 mL/min; Column temperature 40 ℃; Injection Volume 2 μL.

Mass profile conditions: electric spray ion source (ESI) temperature, 500 °C; ion spray voltage (IS), 5500 V (positive ion mode)/−4500 V (negative ion mode). The ion source gas I (GSI), gas II (GSII), and curtain gas (CUR) were set to 50, 60, and 25 psi, respectively, and the collision-induced ionization parameters were set to high. QQ scanning used MRM mode and the collision gas (nitrogen) was set to medium. By further optimizing the clustering potential (DP) and collision energy (CE), the DP and CE of each MRM ion pair were completed. A specific set of MRM ion pairs were monitored at each period based on the metabolites eluted during each period.

### 2.5 Identification and quantification of metabolites

Analyst 1.6.3 software was used to process mass spectrometry data and perform qualitative and quantitative analysis of metabolites as described by Sun et al. (Sun et al., 2022). Qualitative analysis was conducted by comparing accurate precursor ion (Q1) and yield (Q3) values and retention time (RT) and matching them with the self-built database MWDB (MetWare Biological Co., Ltd., Wuhan, China). Quantitative analysis of metabolites was conducted through multiple reaction monitoring (MRM) analysis of QQ using a mass spectrometer to obtain mass spectrometry data of metabolites in different samples. Then, the peak areas of the mass spectra peaks of all metabolites were integrated. Multi quantum (AB SCIEXFramingham, MA, USA) software was used to integrate and calibrate chromatographic peaks and calculate the relative concentration of the corresponding substance in the peak area of each chromatographic peak (Fig. S1).

### 2.6 In vitro biological activity evaluation

Sample extraction. A total of 0.1 g of ground sample powder was added to 10 mL of 70 % methanol, extracted with ultrasound assistance for 1 h, and centrifuged at 12,000 r/min for 2 min, and the supernatant was collected to obtain the sample extraction solution, which were stored at 4 ℃. The extract was diluted 8 times for antioxidant experiments, and the original solution was used for lipase inhibition rate experiments. All results are the average of three parallel experiments.

#### 2.6.1 Antioxidant activity

**DPPH.** DPPH was determined according to a method by Senouwa et al. (Dossou et al., 2022), with modifications. Diluted extract (100 μL) and DPPH solution (0.0404 mg/mL) (100 μL) were added to the 96-well plate. The solutions were reacted under room temperature conditions in a dark environment for 1 h, and the absorbance value was measured at 517 nm using a microplate reader. In the control group, 70 % methanol was used instead of DPPH solution. The blank group consisted of 70 % methanol. The DPPH clearance rate was calculated as follows: DPPH clearance rate = [1 − (sample absorbance − reference absorbance) ÷ blank absorbance] × 100.

**ABTS.** Refer to the Senouwa et al. method for ABTS determination, which was followed with slight modifications (Dossou et al., 2022). The ABTS solution (6.94 mmol/L) was mixed with K_2_S_2_O_8_ solution (2.6 mmol/L) and reacted thoroughly in a cool place for 12–16 h. Then, the mixed solution were diluted 8 times with 70 % methanol (with an absorbance of 7.00 ± 0.02 at 734 nm) to obtain the ABTS working solution. Then, 25 μL of diluted extract was added to the 96-well plate, 175 μL of the working solution of ABTS was mixed well and reacted in the dark for 40 min, and the absorbance value was measured at 734 nm using a microplate reader. In the control group, 70 % methanol was used instead of DPPH solution. The blank group consisted of 70 % methanol. The ABTS clearance rate was calculated as follows: ABTS clearance rate = [1- (sample absorbance - reference absorbance) ÷ blank absorbance] × 100.

**FRAP.** The FRAP clearance rate was determined using a total antioxidant assay kit (Nanjing Jiancheng Bioengineering Research Institute, China). In short, the standard curves for the determination of FeSO_4_ solutions were prepared with concentrations of 0.15, 0.3, 0.6, 0.9, 1.2, and 1.5 mmol/L. Then, 180 μL of FRAP working fluid was added to the 96-well plate, 5 μL of FeSO_4_ solutions of different concentrations and 5 μL of diluted extracts each. In the blank group, the sample extraction solution was replaced with distilled water. The mixed solution were incubated at 37 °C for 3–5 min, and the absorbance value was measured at 593 nm using a microplate reader. The standard curve is: y = 0.3144x + 0.0032 (R^2^ = 0.9994).

#### 2.6.2 Pancreatic lipase (PL) inhibition rate

The inhibitory activity of PL was measured using a colorimetric assay according to Hu et al.'s method (Hu et al., 2015) with slight modifications. Pancreatic lipase was dissolved in 0.1 mol/L Tris-HCl buffer (pH 8.2) and prepared as an enzyme solution of 0.01 g/L. The reaction substrate was 4-nitrophenyl laurate (0.8 mg/mL), which was prepared using a sodium acetate buffer solution (5 mmol/L, pH 5.0). Then, 200 μL of sample extract, 200 μL of pancreatic lipase solution and 500 μL of mixed Tris-HCl buffer solution were added and incubated at 37 °C for 10 min. After reacting with substrate (600 μL) at 37 °C for 20 min, the reaction was terminated by boiling water for 10 min. The blank group contained 700 μL Tris-HCl buffer, 200 μL pancreatic lipase solution and 600 μL substrate. The blank control group and sample control group were treated with buffer solution instead of pancreatic lipase solution, respectively, and orlistat (ORL) was used as a positive control. After the reaction, the sample was centrifuged for 3 min (12,000 r/min), the supernatant was collected, and the absorbance was measured at a wavelength of 405 nm. The formula for calculating the inhibition rate is as follows: inhibition rate/%=[1- (Asample - Asample control)/(Ablank - Ablank control)] × 100 %.

#### 2.6.3 Cultivation and induction differentiation of 3T3L1 pre-adipocytes

According to Wei et al.'s method (Wei et al., 2021), 3 T3-L1 cells were cultured in growth medium (DMEM (high sugar), 10 % FBS, 1 % P/S). For adipocyte differentiation, cells were cultured to confluence and incubated with DMEM (high glucose) plus 10 % FBS, 0.125 mM indomethacin, 1 nMT3, 20 nM insulin, 5 mM dexamethasone, 1 mM rosiglitazone and 0.5 mM Sigma for 2 days. The cells were then maintained in growth medium containing 1 nMT3 and 20 nM insulin for an additional 4 days, was added and replaced every 2 days. By day 6, the mature adipocytes were switched to growth medium and treated with inducer-containing samples at different concentrations for 24 h before harvesting. Model group (MOD), cells induced with inducer only; positive group (ORL), cells incubated with inducer-containing orlistat; experimental group, cells incubated with inducer-containing samples at different maturity stages.

#### 2.6.4 Analysis of 3T3-L1 preadipocyte viability

The effect of CRZP extract on cell viability of 3T3-L1 preadipocytes was determined by CCK8 method (Zheng et al., 2023). The 100 μL 1.5 × 10^5^ cells/mL 3T3-L1 cells were inoculated for 24 h. CRZP extract was prepared at different concentrations (0, 0.5, 1, 1.5, 2, 2.5, 3, 3.5 and 4 mg/mL), added to 3T3-L1 cells separately, and incubated for 24 h. 3T3-L1 cell was washed with 1 × PBS once, added 100 μL serum-free medium and 10 μL CCK8 solution, incubated for 1.5 h, and then measured the absorbance at 450 nm. Cell viability was calculated as: Cell viability (%) = [ (A_1_-A_0_)/(A_2_-A_0_)] × 100 %.

Where A_1_ represents absorbances of cells treated with CRZP, CCK8, and serum-free medium, A_2_ represents absorbances of cells without CRZP treated, but CCK8, and serum-free medium, and A_0_ represents absorbances of CCK8, and serum-free medium, but no cells.

#### 2.6.5 Oil red O staining and observation

Induce differentiation of 3T3-L1 cells using the method described in 2.6.3 and simultaneously add CRZP extract. After cell induction, 1 mL of 4 % paraformaldehyde solution was used to immobilise the cells for 15 min. Then, the cells were washed with PBS twice. The staining process was performed according to the Oil Red O staining kits method, and the lipid droplets condition in the cells and teh cells morphology was recorded under an inverted microscope.

#### 2.6.6 Determination of glycerol and FFA in adipocytes

Evaluate lipolytic activity by detecting intracellular glycerol and Free Fatty Acid (FFA) release (Wei et al., 2021). The fully differentiated adipocytes were cultured in serum-free DMEM for 3 h in a 96-well plate, with or without CRZP. The culture medium from each well was collected for detection glycerol and FFA levels using glycerol assay kit and NEFA assay kit, respectively. Glycerol and FFA levels were normalized to total cellular protein amount using the BCA protein assay kit.

#### 2.6.7 Intracellular ROS, MDA, SOD and GSH content analysis

Treat differentiated cells with CRZP extract for 24 h, then collect the cells and add protease inhibitors in 1:100 ratio to 1 × PBS buffer. Crush cells in an ice water bath to prepare a cell suspension. The protein concentration of cells was detected using a BCA kit, while the ROS, MDA, SOD and GSH content of each group was detected using a ROS assay kit, MDA assay kit, SOD assay kit and GSH assay kit, respectively.

### 2.7 Statistical analysis

All measurements were conducted in triplicates and the results were presented as the mean ± SD. To ensure that the experimental results were accurate, a quality control sample was inserted into every 2 detection and analysis samples, and the total ion flow diagrams of different quality control samples for mass spectrometry detection and analysis were overlapped and displayed for analysis to monitor the repeatability of the analysis process. The statistical function prcomp in R software was used for principal component analysis (https://www.r-project.org/), and the ComplexHetmap package in R software was used for hierarchical cluster analysis (HCA) and to draw heatmaps. The MetaboAnalystR package in R software calculates VIP values, permutations, and rating maps in OPLS-DA and screens differential metabolites using criteria with VIP > 1 and P < 0.05. GraphPad Prism 8.4.3 (GraphPad Software, USA) was used for statistical analysis, plotting bar charts and ANOVA analysis (*p* < 0.05 indicated statistical significance). Pearson correlation was used to analyze the correlation between variables. In addition, the metabolites obtained will be annotated through the KEGG database (compounds, https://www.kegg.jp/kegg/compound/; Pathways, http://www.kegg.jp/kegg/pathway.html).

## 3 Results and discussion

### 3.1 Identification of metabolites from CRZP at different maturation stages

**Fig. 2:**
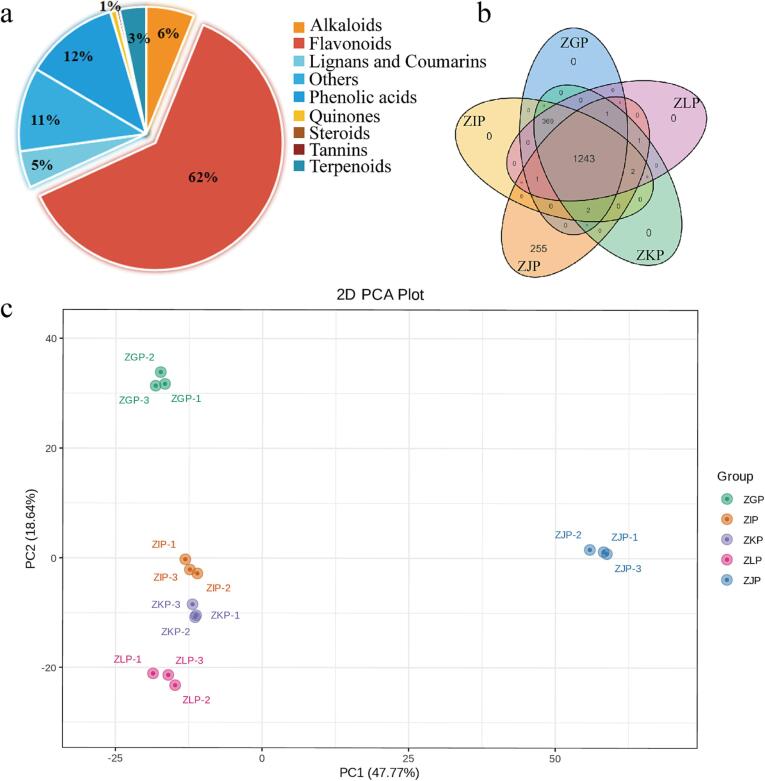
Statistics and differences of metabolites in CRZP at different maturation stages. (a) Classification pie chart of identified metabolites. (b) The distribution of 1,878 metabolites in different ripening stages of Tibetan orange peel. (c) Principal Component Analysis Results Chart. (For interpretation of the references to color in this figure legend, the reader is referred to the web version of this article.)

Citrus peel is a major byproduct of the citrus industry, accounting for approximately half of the total weight of citrus fruits. Due to processes such as microbial decay, citrus peel may become an economic and environmental issue (Mahato et al., 2018); as a result, the development and utilization of citrus peel based on chemical composition research is particularly important. To detect the variability of metabolites in CRZP at different maturity stages, we used a nontargeted metabolomics method based on UPLC–MS/MS and conducted metabolomics analysis on CRZP from five harvesting periods with four maturity stages (the immature stage, the incomplete maturity stage, the commercial ripening stage, and the fully matured stage). The samples were analyzed in negative and positive spray ionization (ESI) modes, which is helpful for detecting more metabolites (Farag et al., 2019). The repeatability and reliability of the method have been confirmed by the quality control (QC) sample results (Farag et al., 2019, Senouwa et al., 2022). Total ion chromatography (TIC) shows the differences in chromatographic specifications (Fig. S2), as well as the repeatability and reliability of the results. The MRM results are shown in Fig. S3, with each peak of different colors representing the metabolites detected in the sample. A total of 1,878 metabolites were identified (Table S1, Fig. 2a, 2b), including 737 flavonoids, 364 phenolic acids, 276 alkaloids, 203 lignans and coumarins, 120 terpenoids, 10 quinones, 6 tannins, 1 steroid, and 161 other types, mainly flavonoids (62.04 %), followed by phenolic acids (11.97 %) and alkaloids (6.11 %). The main active components in CRZP are phenolic acids and flavonoids, which is similar to most citrus varieties, such as *C. reticulata* Blanco. Thus, CRZP exhibits potential antioxidant, anti-inflammatory, and lipid-lowering activities (Deng et al., 2023, Singh et al., 2020).

**Fig. 3:**
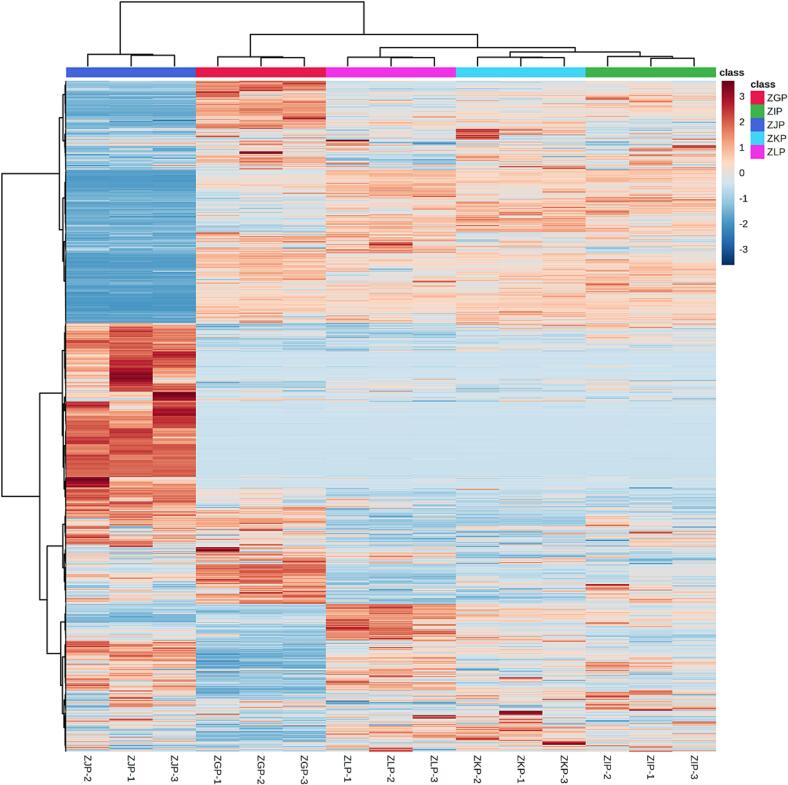
The cluster heat map of metabolites in CRZP at Different Maturation Stages.

In addition, we investigated the effect of maturity on the composition of CRZP components. Our previous research has shown that maturity can affect the composition of volatile organic compounds in CRZP (Wang et al., 2023, Wang et al., 2023), suggesting that maturity should be an important indicator for evaluating the potential application of citrus peel. Principal component analysis (PCA) helps clarify the differences between group samples and the degree of variation within group samples, as shown in Fig. 2c. Clustering of samples from different groups indicates that the metabolic profiles of CRZP vary at different maturity stages. The clustering of samples within the same group indicates a uniform distribution of metabolites within the same group, which confirms the repeatability and reliability of this experiment. To examine the patterns of metabolites in different groups, we conducted cluster heatmap analysis, as shown in Fig. 3. According to the results of PCA (PC1: 47.77 %; PC2: 18.64 %) and HCA, ZJP could cluster separately from the other four groups of samples and showed a relatively high content of some metabolites detected. Further correlation analysis also supports this result (Fig. S4). The clustering of samples within the same group indicates that the metabolic profile of CRZP is influenced by maturity. Previous studies have shown that as maturity increases, the flavonoid content and lipase inhibitory activity of *C. reticulata* ‘Chachi’ show a decreasing trend (Zeng et al., 2018). The research results of Giulia et al. indicate that maturity has an impact on bioactive compounds, such as phenolic acids and flavonoids, as well as antioxidant activity, in the Phegrean Mandarin (*C. reticulata* Blanco) peel (Costanzo et al., 2022). In our study, there were 1,243 common components in the five stages, and compared to the other four stages, 255 metabolites with specificity were found in the CRZP during the commercial ripening stage (ZJP). This may be related to the effect of temperature on the activity of related enzymes (Lehner and Siegmund, 2020, Liu et al., 2023, Liu et al., 2021).

### 3.2 Differential metabolites in CRZP at different maturation stages

To further clarify the impact of maturity on the metabolic products of CRZP and identify differential metabolites, we conducted OPLS-DA (Fig. S5). Based on the results of PCA and HCA, ZJP can be significantly distinguished from the other four groups. Therefore, ZJP was used as the control group and the other four groups were used as the experimental group to analyze the metabolic differences between the different groups. We observed high predictability (Q2) and high fit (R^2^X, R^2^Y) of the model in the following comparative groups: ZJP and ZGP, ZJP and ZIP, ZJP and ZKP, and ZJP and ZLP (Fig. S6), with Q^2^ values of 0.996, 0.993, 0.992, and 0.993, respectively. The criteria of VIP > 1 and P value < 0.05 were used to screen for differential metabolites compared in pairs, and the results were visualized using a volcanic map (Fig. S7). There were 1,069 (699 upregulated) DEGs between ZJP and ZGP, 997 (636 upregulated) DEGs between ZJP and ZIP, 1026 (639 upregulated) DEGs between ZJP and ZKP, and 1041 (712 upregulated) DEGs between ZJP and ZLP. These differential metabolites are mostly phenolic acids, flavonoids, alkaloids, lignans and coumarins, terpenoids, and steroids. The classification of metabolites with significant differences between ZJP and the other four groups (Fig. S8) indicates that ZJP contains more types of alkaloids, quinones, and steroids. These results indicate that maturity has a significant impact on the metabolic profile of Tibetan orange peel, with varying alterations in metabolites.

### 3.3 KEGG pathway annotation of the differential metabolites

**Fig. 4:**
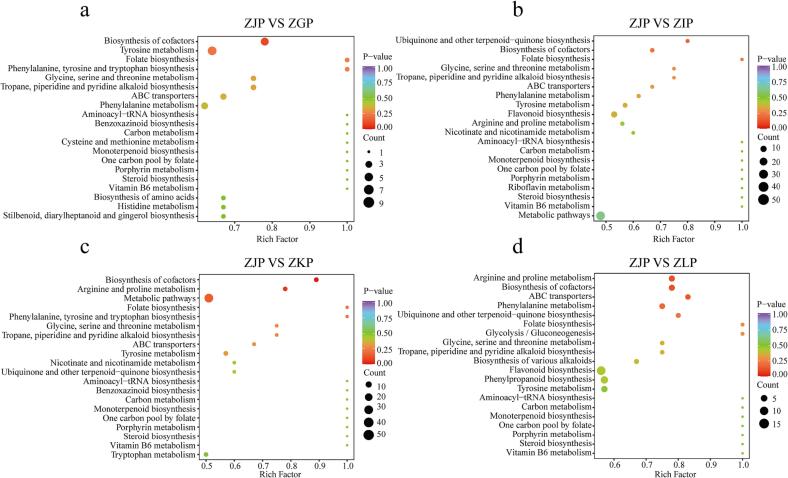
KEGG annotations and enrichment results of the differentially expressed metabolites in the pairwise comparison between: (a): ZJP and ZGP; (b): ZJP and ZIP; (c): ZJP and ZKP; and (d): ZJP and ZLP.

To study differential metabolites at the gene expression level, we conducted KEGG analysis. The annotation results indicate that many differential metabolites are involved in the biosynthesis of cofactors in metabolic pathways, flavonoids, amino acids, and monoterpenoids. KEGG pathway enrichment analysis showed that the differential metabolites between ZJP and ZGP and between ZJP and ZKP were mainly enriched in the biosynthesis of cofactors, amino acids, folate, propane, piperidine, and pyridine alkaloids, carbon metabolism pathways, and monoterpene biosynthesis (Fig. 4a, Fig. 4c). In addition to these metabolic pathways, the differential metabolites between ZJP and ZIP involved flavonoid biosynthesis pathways (Fig. 4b). The differential metabolites between ZJP and ZLP also involved flavonoid biosynthesis and various alkaloid biosynthesis pathways (Fig. 4d).

### 3.4 Key significantly differential metabolites

**Fig. 5:**
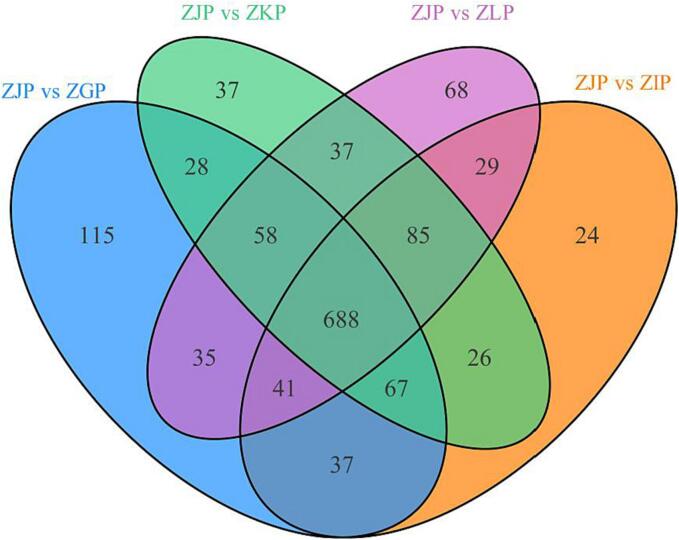
Venn map. The quantity of key differential metabolites in CRZP changes with maturity.

To identify the key differential metabolites that change with increasing CRZP maturity, Venn plots were constructed for ZJP and ZGP, ZJP and ZIP, ZJP and ZKP, and ZJP and ZLP (Fig. 5). The results showed that there were a total of 688 significantly different metabolites among the four paired comparisons. We conducted KEGG analysis on these differential metabolites and annotated a total of 62 key differential metabolites with metabolic pathways, including 21 phenolic acids, 18 flavonoids, 14 alkaloids, 5 lignans and coumarins, 3 terpenoids, and 1 other class (Table S2). Phenolic acids and flavonoids accounted for 34.07 % and 24.09 % of the key metabolites, respectively. As maturity increases, the most biologically active compounds with antioxidant, relieving or treating cardiovascular diseases and antiviral properties are upregulated, such as α-hydroxycinnamic acid, 2-hydroxycinnamic acid, protocatechuic acid, salidroside, 3,4,2′,4′,6′-pentahydroxychalcone, hesperidin, isovitexin, lonicerin, quercetin, butin, 5-aminolevulinic acid, histamine, bergapten, scopoletin and aesculetin (Gomez-Mejia et al., 2019, Maqbool et al., 2023; Tripoli et al., 2007; Yuan et al., 2023), most of which are phenolic acids (34.07 %) and flavonoids (24.09 %). Previous studies have shown that quercetin is a 3C-like protease inhibitor of SARS-CoV-2 that can inhibit SARS-CoV-2 virus (COVID-19) infection (Derosa et al., 2021, Ulusoy and Sanlier, 2020). Hesperidin significantly inhibits IL-1 by inhibiting cellular pathways such as MAPK and JNK β, IL-6 and other inflammatory factors to inhibit the infection of novel coronavirus (Zalpoor et al., 2022). The identified active components laid the foundation for further research on the antioxidant and lipid-lowering activities of CRZP.

### 3.5 Changes in differential metabolites and biological activity of CRZP at different maturation stages

#### 3.5.1 DPPH, ABTS and FRAP

**Fig. 6:**
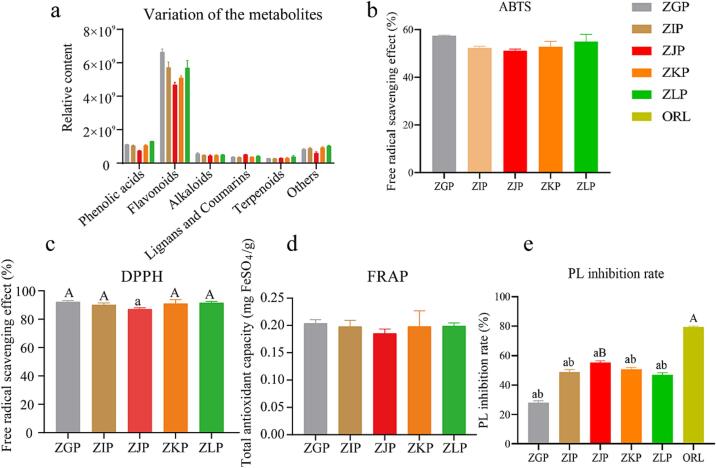
The relative content, antioxidant activity, and PL inhibition rate of various metabolites in different mature stages of CRZP. (a) Comparison of relative contents of various metabolites among the five groups of ZGP, ZIP, ZJP, ZKP, and ZLP; (b) ABTS measurement; (c) DPPH measurement; (d) FRAP method was used to determine the total antioxidant activity of orange peel at different ripening stages; (e) Determination of PL inhibition rate. The same uppercase and lowercase letters on the bar chart indicate statistically significant differences (*p* < 0.05). (For interpretation of the references to color in this figure legend, the reader is referred to the web version of this article.)

The naturally occurring phytochemicals in citrus peel are potential sources of antioxidants and lipase inhibitors (Maqbool et al., 2023, Zeng et al., 2018, Dietrich and Horvath, 2012, Yuan et al., 2023). During the aging process of organisms, unstable reactive oxygen species are produced due to natural physiological processes, nutritional conditions, exposure to sunlight, and other external factors, which affect the function of enzymes in cells (Maqbool et al., 2023, Senouwa et al., 2022). To reveal the potential correlation between the metabolic profile, antioxidant activity, and PL inhibition of CRZP at different ripening stages, we compared the relative content of each type of metabolite in CRZP at different ripening stages. In addition, we evaluated the antioxidant activity of CRZP using DPPH, ABTS, and FRAP methods and evaluated the PL inhibition rate to determine the potential of CRZP as an anti-obesity supplement (Fig. 6). As shown in Fig. 6a, differences were observed in phenolic acids, flavonoids, alkaloids, lignin, and coumarins among ZGP, ZIP, ZJP, ZKP, and ZLP. The content of phenolic acids, flavonoids, and alkaloids in the ZJP group was lower than that in the other groups, while the content of lignans and coumarins was higher than that in the other four groups. As shown in Fig. 6b, 6c, and 6d, the antioxidant activity of CRZP tends to first decrease and then increase with increasing maturity, reaching its lowest value during the commodity maturity period. The DPPH and ABTS results are expressed as the free radical clearance rate (%), and the antioxidant capacity is expressed as milligrams of FeSO_4_ equivalent per gram of CRZP powder (mg FeSO_4_/g weight of CRZP). The results showed that the DPPH clearance rate and ABTS clearance rate of CRZP were 87.12 %–92.25 % and 51.10 %–57.47 %, respectively, and the total antioxidant capacity (FRAP) was 317.08–345.03 mg FeSO_4_/g during the five harvesting periods. The antioxidant capacity of orange peels collected during the immature period was the strongest, while the antioxidant capacity of the products collected during the mature period was the weakest. There was a significant difference in the DPPH clearance rate among the groups (p < 0.05), the trends of the ABTS clearance rate and total antioxidant capacity were consistent, and there was no significant difference between the groups.

#### 3.5.2 Pancreatic lipase inhibition activity

At present, commercially available PL inhibitors represented by orlistat exhibit side effects, and research on safer and more effective PL inhibitors is particularly important. CRZP is frequently added to beef and mutton stews locally to alleviate the greasy taste of meat. Therefore, we also studied the ability of CRZP extract (concentration: 10 mg/ml) to inhibit PL, which was expressed as the PL inhibition rate. The PL inhibitory activity of CRZP is shown in Fig. 6e, and the activity tended to first increase and then decrease, reaching its highest value during the commodity maturity stage. The PL inhibition rate of CRZP during different harvesting periods ranged from 28.04 % to 55.40 %. Interestingly, the PL inhibition rate of the immature and mature stage CRZP were the lowest and highest, respectively, and the latter rate was close to the PL inhibition rate of orlistat. Interestingly, the trend is opposite to the change in antioxidant activity observed. In addition, the ability of mature-stage CRZP to inhibit PL is similar to that of orlistat. Significant differences were found between each group (P < 0.05), and compared to the ZGP group, the other four groups of samples achieved significantly higher inhibition of lipase activity (P < 0.001). The inhibitory ability of the ZJP group on lipase activity was significantly greater than that of the other four groups. Overall, the immature stage of CRZP exhibited the highest antioxidant activity and the lowest PL inhibition rate, while during the mature period of the product, the PL inhibition rate was the highest and the antioxidant capacity was the lowest. These results indicate that maturity has a significant impact on the biological activity of CRZP.

#### 3.5.3 Cell viability

**Fig. 7:**
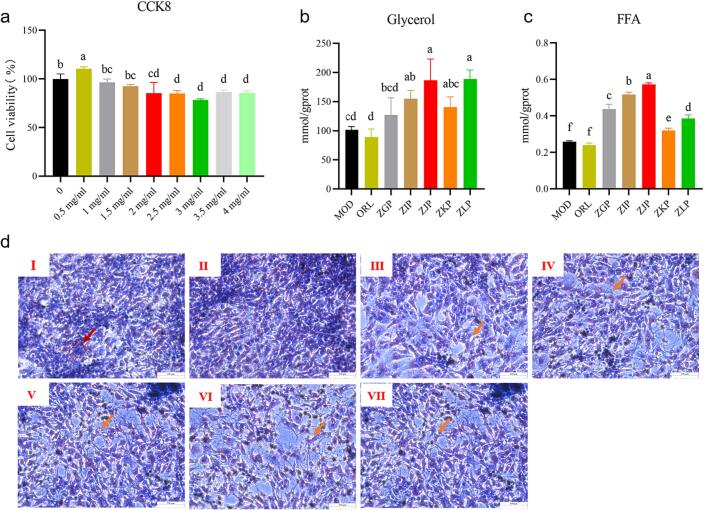
Effect of CRZP on inducing differentiation of 3T3-L1 preadipocytes; (a) Cell viability; (b) Glycerol content; (c) FFA content; (d) Oil Red O Staining images. The red arrow represents lipid droplet accumulation, while the orange arrow represents differentiation inhibition, Ⅰ-Ⅶ represent respectively: MOD, OPL, ZGP, ZIP, ZJP, ZKP, ZLP group. The different lowercase letters in the bar chart represent significant differences between different CRZP at the same concentration. (For interpretation of the references to color in this figure legend, the reader is referred to the web version of this article.)

Fig. 7a showed the influence of CRZP extract on cell viability of 3T3-L1 preadipocytes. The results showed that cytotoxicity occurred when the concentration of CRZP extract was 2 mg/mL, where cell viability was 91.77 % ± 0.72 %.

#### 3.5.4 Effect of inducing differentiation of 3T3-L1 preadipocytes

The 3T3-L1 preadipocytes differentiate into adipocytes through induction and complete lipid accumulation during this process (Zheng et al., 2023). Lipid droplets can be stained red with Oil Red O dye. As shown in Fig. 7d, the model group cells showed large red spots, indicating the presence of significant lipid deposition within the cells. After adding CRZP extract, the differentiation of 3T3-L1 preadipocytes was inhibited to varying degrees, with reduced intracellular fat accumulation, smaller lipid droplets, and weaker Oil Red O staining compared to the model group.

#### 3.5.5 Determination of glycerol and FFA

Lipolysis is a catabolic process that hydrolyzes TG stored in lipid droplets liberating glycerols and FFA (Tan et al., 2016). In this study, we investigated the lipolytic effect of CRZP extract on 3T3-L1 adipocytes. As shown in Fig. 7b and 7c, CRZP extract can increase the content of extracellular FFA and glycerol, which indicate that CRZP extract has a promoting effect on adipocyte lipolysis. The ZLP group had the highest content of FFA, while the ZGP, ZIP, and ZJP groups had significantly higher levels of FFA and glycerol than the ZKP group.

#### 3.5.6 Effects of CRZP on intracellular ROS, MDA, SOD and GSH content

**Fig. 8:**
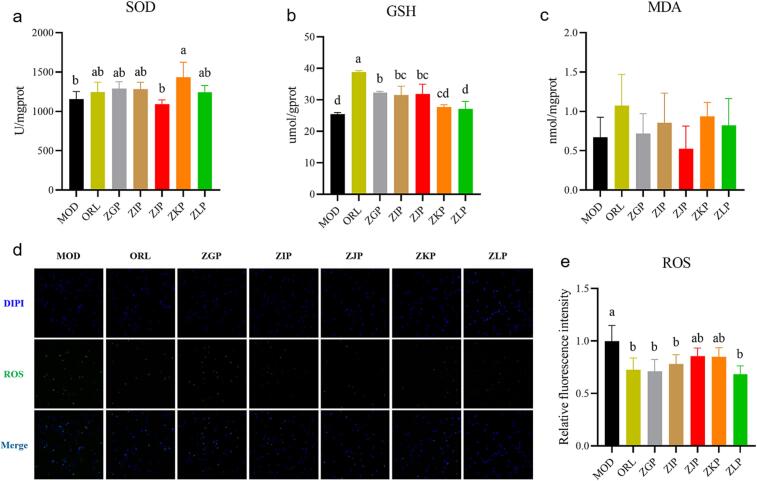
Effect of CRZP on intracellular antioxidant enzyme in 3T3-L1 cells: (a) SOD content; (b) GSH content; (c) MDA content; (d) The intracellular ROS in 3T3-L1 cells after fluorescence staining was exhibited by the image; (e) The relative intensity of fluorescence in different groups. The different lowercase letters in the bar chart represent significant differences between different CRZP at the same concentration..

Oxidative stress plays an important role in the occurrence and development of obesity and its metabolic complications (Li et al., 2022). In the current study, we detected important indicators related to oxidative stress such as ROS, MDA, SOD, and GSHin 3T3-L1 adipocytes, as shown in Fig. 8. CRZP extract can significantly reduce MDA and SOD (Fig. 8c, 8e), and in addition, MDA in the ZJP group is significantly lower than that in the ORL group. The CRZP extract significantly improves oxidative stress damage in 3T3-L1 cells. In addition, the intracellular ROS content in the ZKP and ZLP groups was lower than that in the other three groups, indicating better antioxidant capacity in the fully mature stage. As shown in Fig. 8a and 8b, compared with the MOD group, the content of SOD and GSH in the CRZP sample significantly increased, which is close to the results of the ORL group. It indicates that CRZP could clear intracellular ROS and MDA, while stimulate the body to produce more antioxidant enzymes and has good antioxidant capacity.

#### 3.5.7 Correlation analysis

**Fig. 9:**
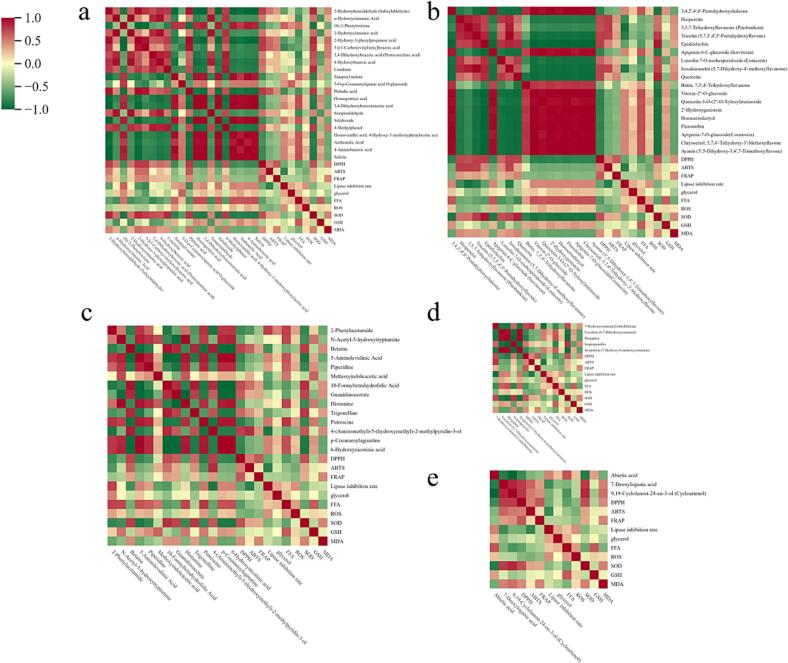
Pearson correlation analysis heatmap of various key differential metabolites in CRZP at different maturity stages. (a) Phenolic acids; (b) Flavonoids; (c) Alkaloids; (d) Lignins and coumarins; (e) Terpenoids.

To investigate the correlation between key differential metabolites and differences in antioxidant activity and lipid-lowering activity, we conducted Pearson correlation analysis, and the results are shown in Fig. 9. Among phenolic compounds (Fig. 9a), phthalic acid, 2-hydroxy-3-phenylpropanoic acid and salicylaldehyde, showed a moderate positive correlation with DPPH, SOD, MDA GSH and FFA (0.3 < | r | < 0.8, r > 0); salicylic acid, protocatechuic acid and salidroside, showed a moderate negative correlation with DPPH, SOD, MDA GSH and FFA(0.3 < | r | < 0.8, r < 0). Phthalic acid and salicylaldehyde showed a moderate positive correlation with ABTS. A moderate negative correlation was found between salidroside and ABTS. Phthalic acid and salicylaldehyde showed a moderate positive correlation with the PL inhibition rate, and salidroside had a moderate negative correlation with the PL inhibition rate, and 5-O-p-Coumaroylquinic acid O-glucoside showed a moderate positive correlation with glycerol. Among the flavonoid compounds (Fig. 9b), epicatechin, pentahydroxyflavone, quercetin and hesperidin, showed a moderate positive correlation with DPPH, SOD, MDA and GSH; lutein, showed a moderate negative correlation with DPPH and MDA. There is a moderate positive correlation between hesperidin, pinobanksin, pentahydroxyflavone and isosakurarin with ABTS, MDA and SOD. There is a moderate negative correlation between hesperidin, quercetin, pentahydroxyflavone and epicatechin with PL inhibitory activity and FFA.

Alkaloid compounds (Fig. 9c) showed a moderate positive correlation with DPPH, MDA and SOD, including 5 compounds such as betaine and cucurbitine; 8 compounds, such as histamine, putrescine and 6-hydroxynicotinic acid, showed a moderate negative correlation with DPPH, MDA and ROS. Betaine and 10-formyltetrahydrofolate showed a moderate positive correlation with ABTS; putrescine and 2-phenylacetamide were moderately negatively correlated with ABTS. 2-Phenylacetamide and putrescine showed a moderate positive correlation with PL inhibitory activity, FFA and glycerol; 5 compounds, such as betaine, showed a moderate negative correlation with the PL inhibition rate, FFA and glycerol. In lignin and coumarin compounds (Fig. 9d), there was a moderate positive correlation between umbelliferone and isoanisole lactone with DPPH, MDA and SOD; aesculetin, scopoletin and bergapten showed a moderate negative correlation with DPPH, MDA and SOD. Umelliferone showed a moderate positive correlation with ABTS and a strong negative correlation with the PL inhibition rate (0.8 < | r |, r < 0). A moderate positive correlation was found between aesculin and scopoletin and the PL inhibition rate, FFA and FFA and glycerol. These results are similar to previous studies (Dadwal et al., 2022, Fardoun et al., 2020, Singh et al., 2020).

## 4 Conclusion

In this study, we used nontargeted metabolomics technology based on UPLC–MS/MS and measured the antioxidant activity and PL inhibition rate to study CRZP at different maturity stages. The goal of this study was to evaluate the potential and possibility of CRZP as a natural functional food source, as well as the impact of maturity on the metabolic characteristics and biological activity of CRZP. A total of 1,878 metabolites were identified. The main metabolites were flavonoids (62.04 %), followed by phenolic acids (11.97 %) and alkaloids (6.11 %). PCA, HCA, and OPLS-DA were used to analyze the metabolic differences of different ripening stages of CRZP, and significant changes were observed in the metabolic profiles of secondary metabolites of CRZP during the commercial ripening period. Furthermore, the key active compounds significantly affected by maturity in CRZP were screened by constructing a Venn map, and the Pearson correlation coefficient was used to analyze the correlation between these compounds and antioxidant activity and lipid-lowering activity. Compounds with moderate correlation with antioxidant activity and lipid-lowering activity, such as protocatechuic acid, salidroside, quercetin, hesperidin, betaine, and cucurbitine, were screened out. Overall, this study reveals a potential correlation between key active ingredients during the ripening process of CRZP and the antioxidant activity and lipid-lowering abilities. Thus, CRZP is natural antioxidants and possess anti-obesity potential, and industrial production needs to consider the mature stage of its collection.

## CRediT authorship contribution statement

**Peng Wang:** Conceptualization, Data curation, Writing – original draft. **Haifan Wang:** Formal analysis. **Yang Xiao:** Visualization. **Jialiang Zou:** Data curation, Software. **Hongping Chen:** Methodology, Conceptualization. **Lin Chen:** Methodology. **Fu Wang:** Writing – review & editing. **Yuan Hu:** Investigation, Project administration. **Youping Liu:** Funding acquisition, Resources, Supervision, Validation.

## Declaration of competing interest

The authors declare that they have no known competing financial interests or personal relationships that could have appeared to influence the work reported in this paper.

## Data Availability

No data was used for the research described in the article.
