# Supplementary material for: Insights into metabolic characteristics and biological activity changes in Zangju (Citrus reticulata cv. Manau Gan) peel at different maturity stages through UPLC–MS/MS-based metabolomics
Source: Food Chem X. 2024 Feb 5;21:101197. doi: 10.1016/j.fochx.2024.101197 (PMC10865237; doi:10.1016/j.fochx.2024.101197)
Supplement: Supplementary data 1 [file mmc1.docx]

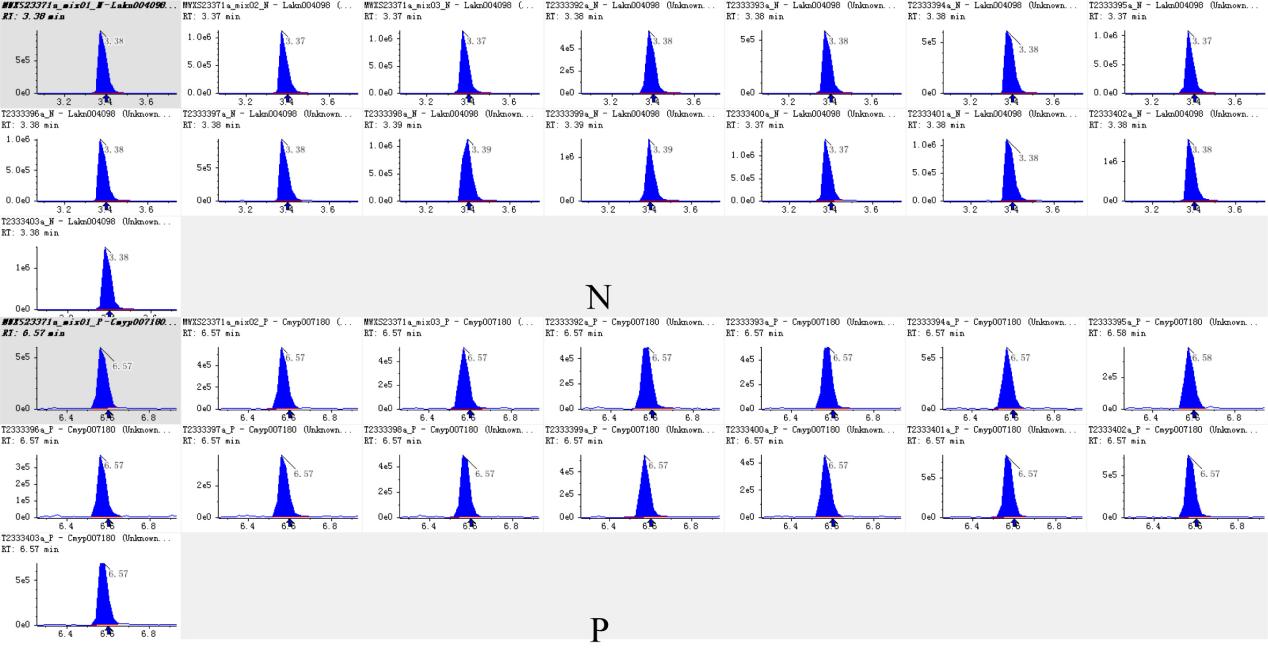


**Figure S1.** Integral correction diagram for quantitative analysis of some of the detected metabolites. The abscissa is the retention time (min) of the metabolite detection. The ordinate is the ion flow strength (CPS)detected by a metabolite ion. The peak area represents the relative content of the substance in the sample.


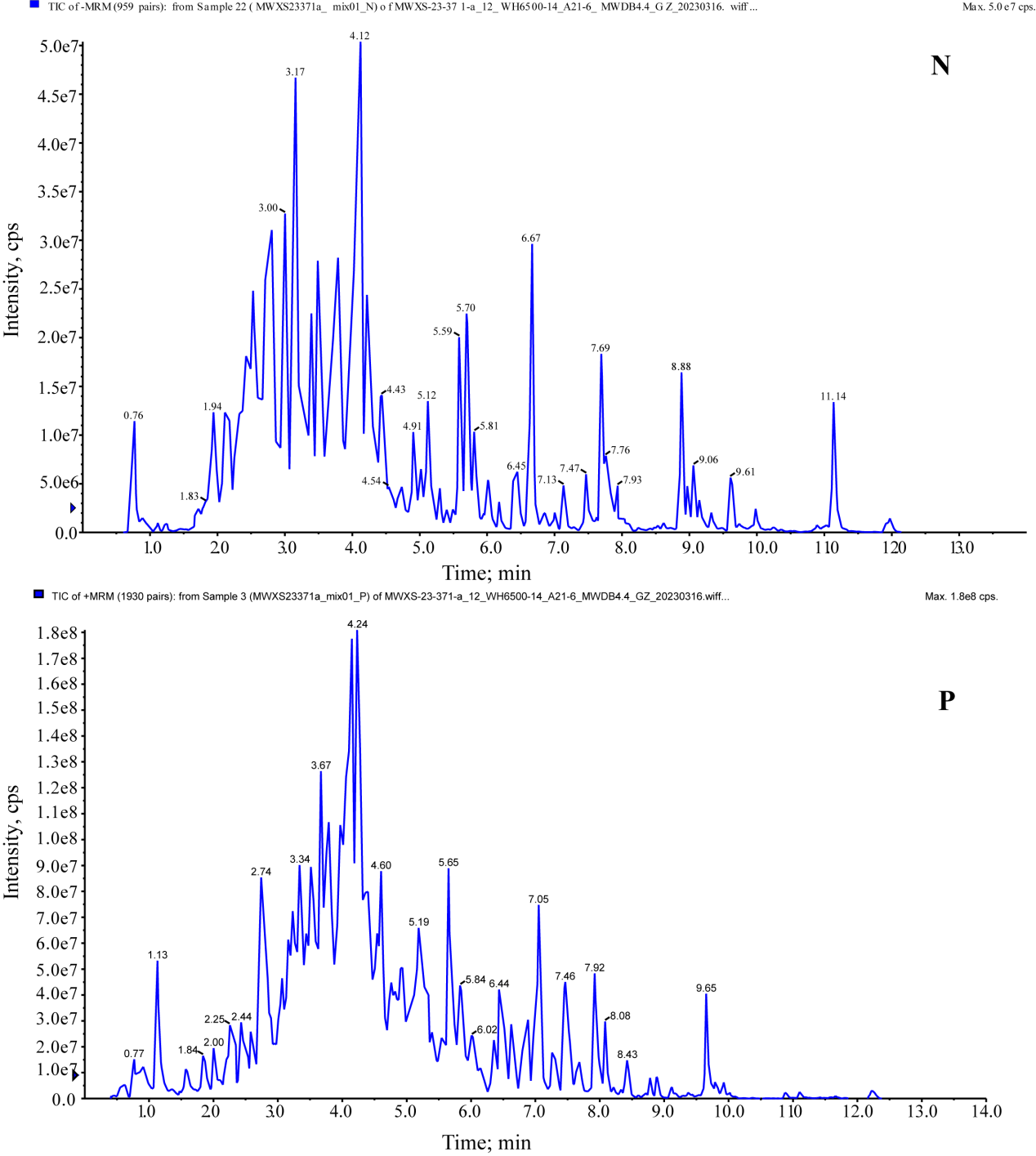


**Figure S2.** Total ions current (TIC) overlapping map of QC samples results. Note: The abscissa is the retention time of the metabolite. The ordinate is the ion current intensity of the ion detection (the intensity units are counts per second (cps)). N stands for negative ion mode; P stands for positive ion mode.


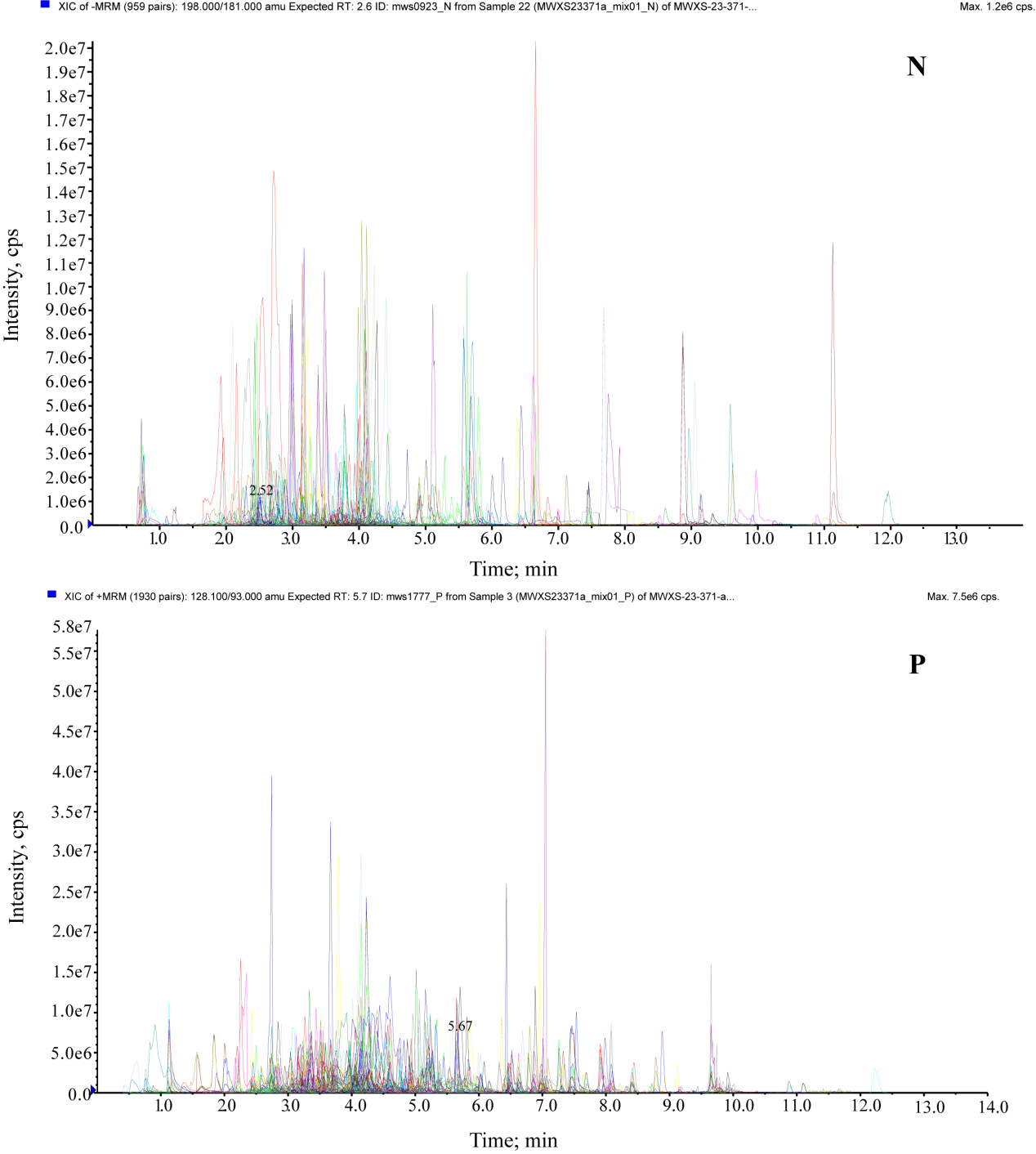


**Figure S3.** Multiple reaction monitoring (MRM) graph of the QC sample. Note: Each color indicates a detected metabolite in the sample. The abscissa is the retention time of the metabolite. The ordinate is the ion current intensity of the ion detection (the intensity units are counts per second (cps)). N stands for negative ion mode; P stands for positive ion mode.


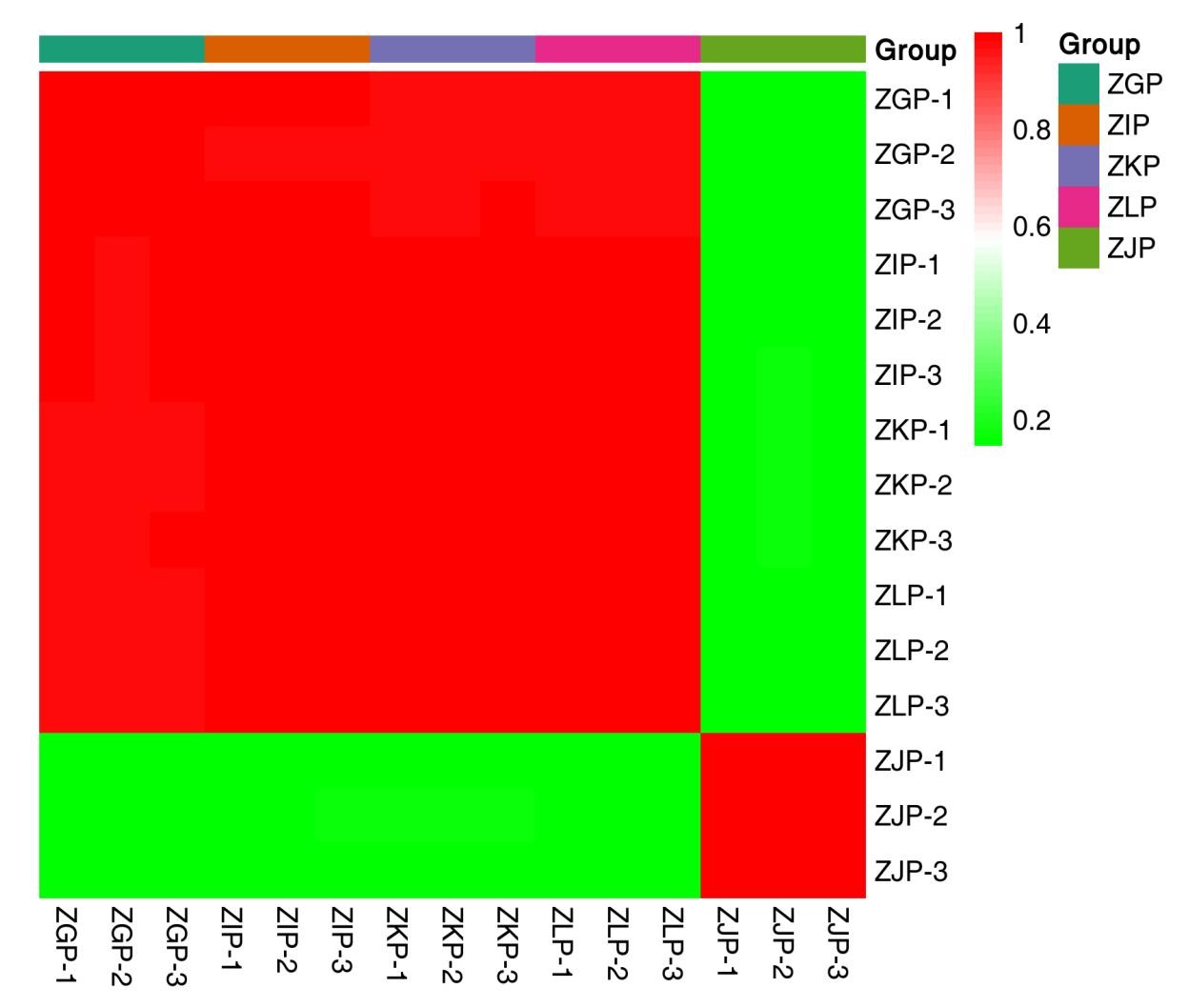


**Figure S4.** The heatmap of the correlations between samples.


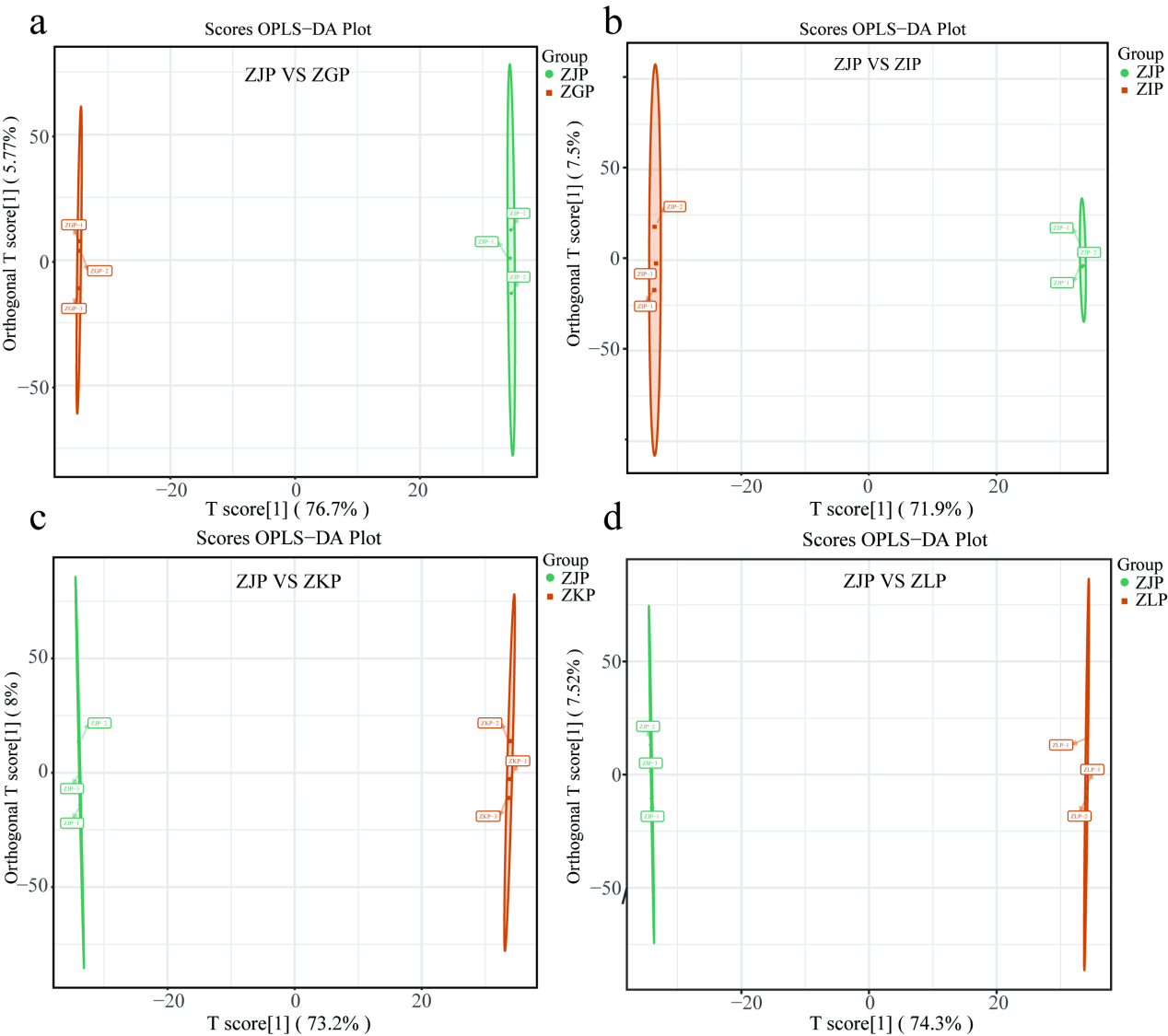


**Figure S5.** The score plots of OPLS-DA pairwise comparisons of metabolites between: (a) ZJP and ZGP; (b) ZJP and ZIP; (c) ZJP and ZKP; (d) ZJP and ZLP.


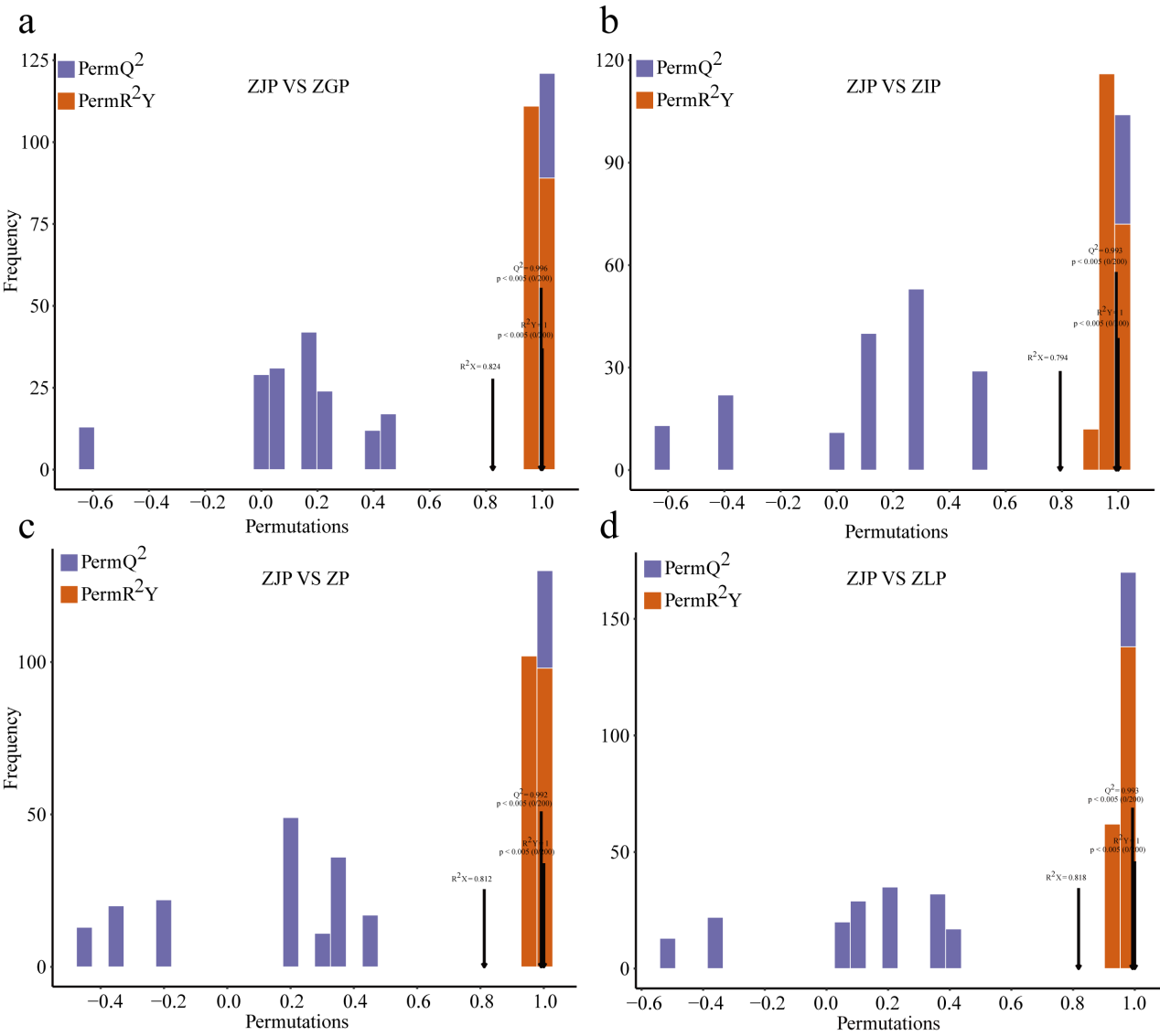


**Figure S6.** OPLS-DA verification diagram of the pairwise comparison of metabolites between: (a) ZJP and ZGP; (b) ZJP and ZIP; (c) ZJP and ZKP; (d) ZJP and ZLP.


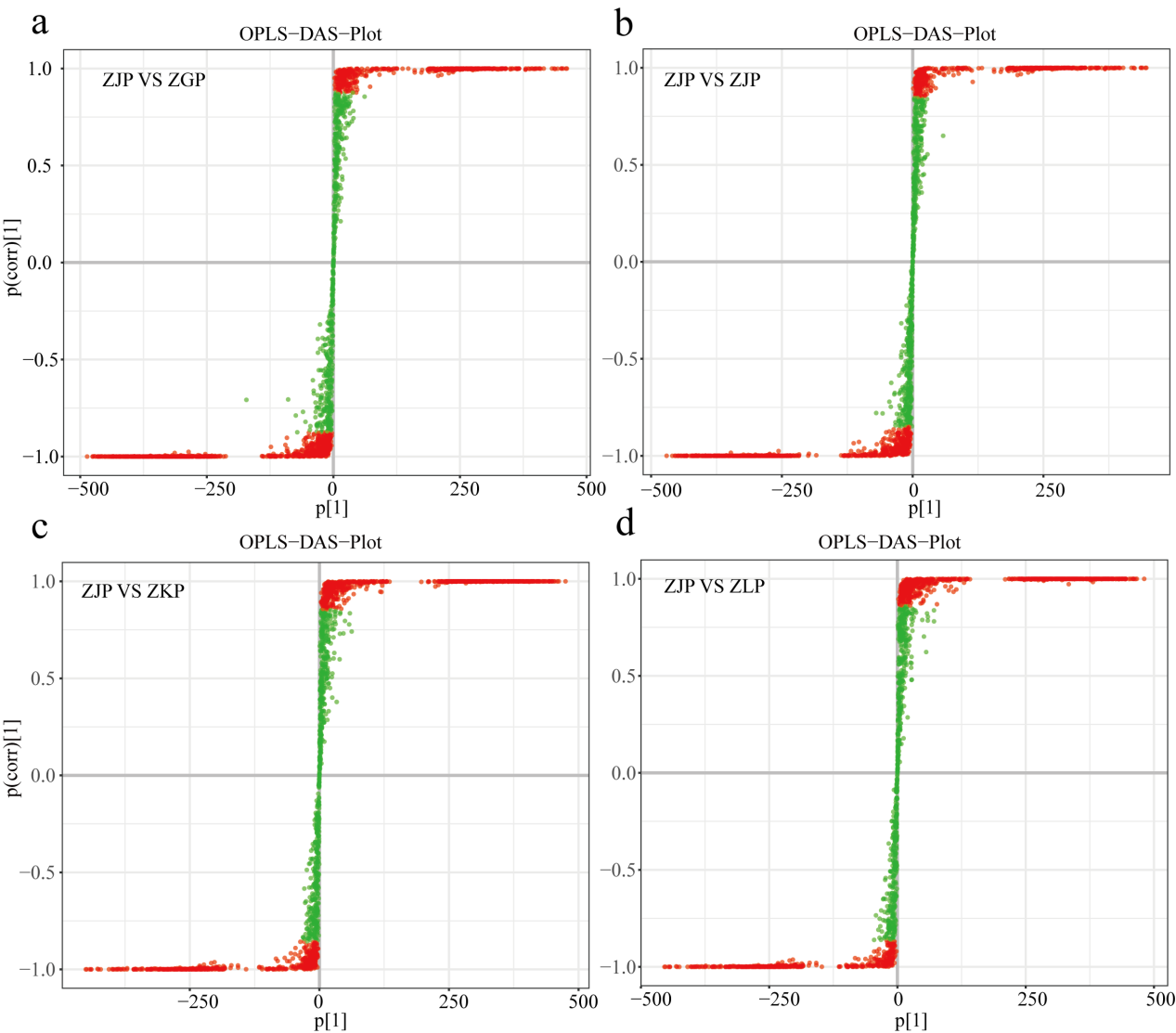


**Figure S7.** Volcano plots of differential metabolites in the pairwise comparison between:(a) ZJP and ZGP; (b) ZJP and ZIP; (c) ZJP and ZKP; (d) ZJP and ZLP. Note: Each point in the volcano map represents a metabolite, the abscissa represents the logarithmic value of the difference of relative content of a certain metabolite in the two samples, the ordinate represents the VIP value. The greater the absolute value of the abscissa, the greater the multiple difference in the expression level between the two samples; the greater the ordinate value, the more significant the differential expression, and the more reliable the differentially expressed metabolites screened. In the figure, the green dots represent down-regulated differentially metabolites, the red dots represent up-regulated differentially metabolites, and gray represents detected but not significantly different metabolites.
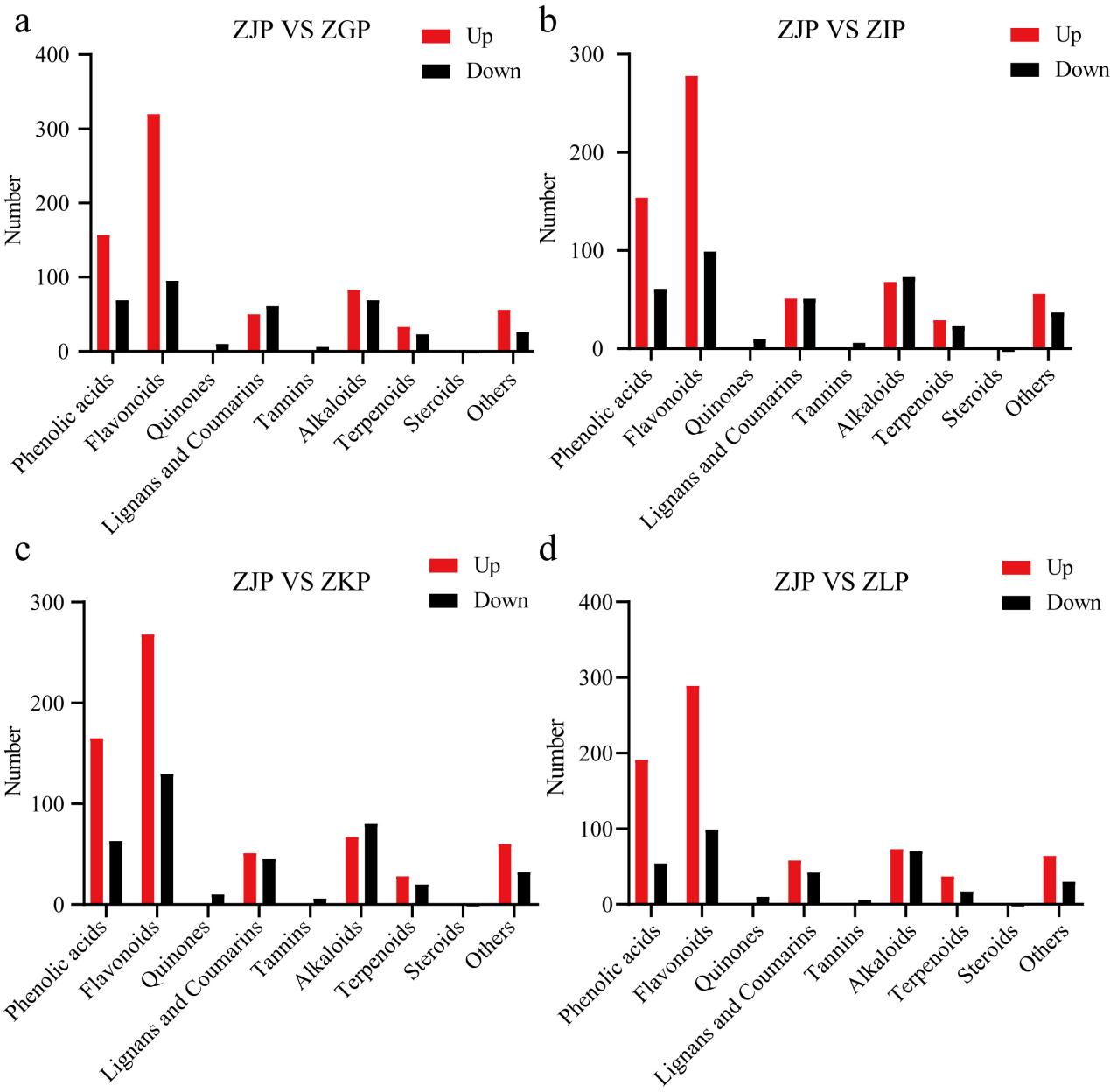


**Figure S8** Classification of the differential metabolites in the pairwise comparison between: (a) ZJP and ZGP; (b) ZJP and ZIP; (c) ZJP and ZKP; (d) ZJP and ZLP.
